# Investigating gender-specific effects of familial risk for attention-deficit hyperactivity disorder and other neurodevelopmental disorders in the Swedish population

**DOI:** 10.1192/bjo.2020.47

**Published:** 2020-06-18

**Authors:** Joanna Martin, Laura Ghirardi, Qi Chen, Catharina A. Hartman, Mina A. Rosenqvist, Mark J. Taylor, Andreas Birgegård, Catarina Almqvist, Paul Lichtenstein, Henrik Larsson

**Affiliations:** MRC Centre for Neuropsychiatric Genetics and Genomics, Cardiff University, UK; and Department of Medical Epidemiology & Biostatistics, Karolinska Institutet, Sweden; Department of Medical Epidemiology & Biostatistics, Karolinska Institutet, Sweden; Department of Medical Epidemiology & Biostatistics, Karolinska Institutet, Sweden; Department of Psychiatry, University Medical Center Groningen, University of Groningen, the Netherlands; Department of Medical Epidemiology & Biostatistics, Karolinska Institutet, Sweden; Department of Medical Epidemiology & Biostatistics, Karolinska Institutet, Sweden; Department of Clinical Neuroscience, Karolinska Institutet, Sweden; Department of Medical Epidemiology & Biostatistics, Karolinska Institutet, Sweden; and Pediatric Allergy and Pulmonology Unit at Astrid Lindgren Children's Hospital, Karolinska University Hospital, Sweden; Department of Medical Epidemiology & Biostatistics, Karolinska Institutet, Sweden; Department of Medical Epidemiology & Biostatistics, Karolinska Institutet; and School of Medical Sciences, Örebro University, Sweden

**Keywords:** Attention deficit hyperactivity disorders, anxiety disorders, depressive disorders, bipolar affective disorders, eating disorders

## Abstract

**Background:**

Many psychiatric disorders show gender differences in prevalence. Recent studies suggest that female patients diagnosed with anxiety and depression carry more genetic risks related to attention-deficit hyperactivity disorder (ADHD) compared with affected males.

**Aims:**

In this register-based study, we aimed to test whether female patients who received clinical diagnoses of anxiety, depressive, bipolar and eating disorders are at higher familial risk for ADHD and other neurodevelopmental disorders, compared with diagnosed male patients.

**Method:**

We analysed data from a record-linkage of several Swedish national registers, including 151 025 sibling pairs from 103 941 unique index individuals diagnosed with anxiety, depressive, bipolar or eating disorders, as well as data from 646 948 cousin pairs. We compared the likelihood of having a relative diagnosed with ADHD/neurodevelopmental disorders in index males and females.

**Results:**

Female patients with anxiety disorders were more likely than affected males to have a brother with ADHD (odd ratio (OR) = 1.13, 95% CI 1.05–1.22). Results for broader neurodevelopmental disorders were similar and were driven by ADHD diagnoses. Follow-up analyses revealed similar point estimates for several categories of anxiety disorders, with the strongest effect observed for agoraphobia (OR = 1.64, 95% CI 1.12–2.39). No significant associations were found in individuals with depressive, bipolar or eating disorders, or in cousins.

**Conclusions:**

These results provide modest support for the possibility that familial/genetic risks for ADHD may show gender-specific phenotypic expression. Alternatively, there could be gender-specific biases in diagnoses of anxiety and ADHD. These factors could play a small role in the observed gender differences in prevalence of ADHD and anxiety.

## Background

Certain psychiatric disorders are diagnosed more commonly in male patients (for example attention-deficit hyperactivity disorder (ADHD) and other neurodevelopmental disorders, such as autism),^1,2^ or in female patients (for example anxiety, depression, eating disorders and to a lesser extent also bipolar disorders).^3–7^ The reasons for these widespread gender differences in prevalence are unknown. Not only are psychiatric disorders moderately to highly heritable, there is also growing evidence of a substantial component of shared genetic risks across many disorders.^8,9^ It is plausible that the genetic liability for psychiatric problems could manifest in a gender-specific manner, partly explaining the observed prevalence differences.

Indeed, in children diagnosed with anxiety or depression (both female-biased disorders), girls have on average a higher burden of common (i.e. polygenic risk) and rare (i.e. copy number variant) genetic risks implicated in ADHD (a male-biased disorder).^10,11^ These findings support the possibility of gender-specific manifestation of biological risk, where the same risk variants are associated with different psychiatric disorders in males (for example ADHD) and females (for example anxiety/depression). However, these studies included fewer than 400 affected children, many of whom were young, not having passed through the risk period for depression, which limited the generalisability of the results to early-onset anxiety and depression.

## Aims

In the current study, our aim was to examine a much larger population sample of children and adults, clinically diagnosed with a wider range of psychiatric disorders (male-biased: ADHD and other neurodevelopmental disorders; female-biased: anxiety, depressive, bipolar and eating disorders). We used a different design to that used in previous research, to test for converging evidence using diverse approaches with different strengths and limitations. We used information from a national population register to define diagnoses based on diagnostic codes from specialist in-patient and out-patient records to examine potential gender-specific effects in real-life diagnostic practices. We used nationwide data on biological relatives to define genetic liability for psychiatric disorders based on familial risk (which encompasses both common and rare inherited variants). We hypothesised that female patients who have received clinical diagnoses of anxiety, depressive, bipolar and eating disorders are at higher familial risk for ADHD and other neurodevelopmental disorders, compared with male patients who have received these diagnoses.

## Method

### Cohort and variable definition

We used a data linkage of several Swedish registers based on each individual's personal identification number.^12^ The linkage was approved by the regional ethics review board in Stockholm, Sweden. The requirement for informed consent was waived because the study was register based and individuals were not personally identifiable. We used information from the Medical Birth Register,^13^ Total Population Register,^14^ National Patient Register (NPR),^15^ Multi-Generation Register^16^ and Cause of Death Register.^17^ We identified all individuals who were living in Sweden and were born between 1964 and 2008, and had received an ICD-10 diagnosis in any of the four categories of psychiatric disorders of interest (i.e. anxiety, depressive, bipolar or eating disorders)^18^ between 1997 and 2013. These dates were selected as the NPR was launched in 1964, the ICD-10 was in use from 1997 and follow-up was until the end of 2013, meaning all index individuals were at least 5 years old. To control for changing diagnostic practices over time, we only considered diagnoses based on ICD-10 and not previous versions of the ICD for the four diagnostic categories of interest. Thus, index patients were identified based on available information from 1997 to 2013, when they were aged between 5 and 49 years old.

We retained only index individuals with both known biological parents linked in the Multi-Generation Register and at least one full sibling (excluding twins) or first cousin born between 1987 and 2008. These dates were selected to capture all relatives who would have been children during the usage of the ICD-9 (1987–1997)^19^ and the ICD-10 (1997 onwards) in Sweden and would be at least 5 years old by the end of the available follow-up period. Full siblings were identified based on having the same biological parents but not being born in the same month. First cousins were identified based on having the same maternal or paternal biological grandparents but not the same parents.

Data on clinical diagnoses were obtained from the Swedish NPR, which contains information on in-patient psychiatric care (1987–2013) and specialist out-patient consultations with doctors (2001–2013). It includes specialist diagnoses according to ICD-9 and ICD-10, with ongoing updates and validation of the NPR.^15^ The majority of the diagnoses were obtained from only out-patient consultations (77%) or a combination of in- and out-patient information (93.5%). To improve identification of individuals diagnosed with eating disorders without recorded diagnoses in the NPR, diagnoses were also obtained from the Cause of Death Register (based on ICD-10 codes) and the treatment quality registers (based on DSM-IV criteria)^20^. See Table 1 for diagnostic definitions.

**Table 1:** Descriptive summary of diagnostic categories

Diagnostic category	Details	ICD codes^a^	Exclusion criteria
Anxiety disorders	Social anxiety, phobias, generalised anxiety, panic disorders, separation anxiety, other childhood-onset anxiety disorders	F40, F41, F93	Only diagnosed age <5 years
Major depressive disorders	Single and recurrent major depressive disorders	F32, F33, F34	Only diagnosed age <10 years
Bipolar disorders	Mania, bipolar affective disorder	F30, F31	Only diagnosed age <10 years
Eating disorders	Anorexia nervosa, bulimia nervosa, unspecified eating disorder	F50.0–F50.3, F50.9	Only diagnosed age <8 years
Attention-deficit hyperactivity disorder	Hyperkinetic disorder	314/F90	Only diagnosed age <1 year
Neurodevelopmental disorders	Attention-deficit hyperactivity disorder, autism spectrum disorder, motor disorders, tic disorders, intellectual disability, communication disorders, specific learning disorders, dyslexia	314/F90; 299/F84; 315E, 307D/F82, F984; 307C/F95; 317–319/F70–F79; 315D/F80; 315A/F81; 315B/R48	Only diagnosed age <1 year

a. ICD-10, except for ADHD/neurodevelopmental disorders where both ICD-9 and ICD-10 were used, and except eating disorders where DSM-IV was also used (see main text for details).

For primary analyses, specific diagnoses for female-biased psychiatric disorders were collapsed into four binary variables: any anxiety disorder, any major depressive disorder (MDD), any bipolar disorder and any eating disorder. This was deemed justifiable given the ICD groupings, as well as evidence of diagnostic fluidity, comorbidity and shared genetic influences across diagnostic subtypes.^3,21–24^ Given that the typical onset of these disorders is in mid-childhood/early adolescence, we excluded index individuals who only received diagnoses at a very young age and never again (anxiety disorder: age <5 years; MDD/bipolar disorders: age <10 years; eating disorders: age <8 years). Using these cut-offs, *n* = 575 unique index individuals were excluded from sibling-pair analyses and *n* = 507 from cousin-pair analyses. We also defined variables for age at first recorded diagnosis for each disorder category.

Information on presence of ADHD and neurodevelopmental disorders was also obtained from the NPR for both index individuals and their relatives (i.e. siblings and cousins). A broader group of neurodevelopmental disorders was defined as presence of one or more of the following disorders: ADHD, autism spectrum disorder, motor disorders, tic disorders, intellectual disability, communication disorders, specific intellectual disorders and dyslexia. Both ICD-9 and ICD-10 codes were used (see Table 1) to decrease the false negative rate of exposure to familial risk in index individuals. Binary variables were derived for ADHD and for ‘any neurodevelopmental disorder’. Any individuals who received diagnoses of ADHD/neurodevelopmental disorders only prior to age 1 and never again were assumed to not have ADHD/neurodevelopmental disorders.

### Data analyses

Logistic regression analyses were used to test for an association between familial risk of ADHD/neurodevelopmental disorders and the gender of index individuals diagnosed with anxiety disorders, MDD, bipolar disorders or eating disorders. The exposures were presence of diagnosed: (a) ADHD or (b) any neurodevelopmental disorders, in the sibling (coded as absent, 0 and present, 1) and the dependent variable was the gender of the diagnosed individual (coded as male, 0 and female, 1). All analyses were stratified by the gender of the sibling, to prevent confounding from potential gender-specific diagnostic biases in relatives. These primary analyses were adjusted for multiple testing using a stringent Bonferroni correction (0.05/16 tests); the main results are evaluated against a threshold of *P* < 0.0031.

As the data included non-independent observations, a sandwich estimator was used to cluster the observations by family and derive robust standard errors. The following covariates were included in analyses: birth year of the index individual, birth year of the relative, age at first diagnosis of the disorder of interest (anxiety disorders, MDD, bipolar or eating disorders) in the index individual, and presence of comorbid ADHD/neurodevelopmental disorders (depending on the exposure) in the index individual. All analyses were run using Stata version 15.1.

#### Secondary analyses

Follow-up analyses were used to determine whether results for the broader group of neurodevelopmental disorders differed when ADHD was not considered as part of this broad group. Next, we stratified the index individuals based on presence of comorbid ADHD, to determine whether the results differed in individuals with comorbid diagnoses (who may have a more complex clinical presentation and different genetic liability), compared with those without comorbid ADHD.

For significant primary associations, follow-up analyses tested whether any specific ICD diagnostic codes were driving observed association signals.

To test whether the degree of familial relatedness had an impact on the results, we analysed observations from cousin pairs. Full siblings share on average 50% of their genomes whereas first cousins share 12.5% of their genomes and siblings share a greater degree of their environmental exposures than cousins do, so familial effects are expected to be stronger in siblings.

#### Sensitivity analyses

Several sensitivity analyses were conducted. First, we used more stringent diagnostic definitions to exclude index individuals with only one recorded diagnosis within each category (anxiety disorders, MDD, bipolar and eating disorders). ADHD diagnoses were also only considered as present if they were diagnosed at least twice. This allowed us to investigate whether diagnostic uncertainty could explain the observed results. Second, we excluded all individuals who were aged <18 years at the end of the follow-up period (i.e. those born in 1996 or later) to ensure that all individuals had lived through the main risk period of getting diagnosed with ADHD. Third, the analyses were repeated including only siblings who were born in Sweden and still living there by the end of the follow-up period (end of 2013). Observations from siblings who had migrated or died were excluded. This was done to account for possible uncertainty and missing information during the available follow-up period. See supplementary Table 1 available at https://doi.org/10.1192/bjo.2020.47 for details of sample sizes after applying these exclusions.

## Results

The primary cohort included a total of 103 941 (64.3% female) unique index individuals who were affected with at least one of the female-biased psychiatric disorders of interest and had at least one full sibling; each sibling pair was treated as one observation, yielding 151 025 sibling pairs. For the analyses of cousin pairs, the total sample consisted of 646 948 cousin-pair observations from 171 247 unique index individuals (63.4% female; supplementary Table 1).

As expected, there were more females in the primary cohort for each of the diagnostic categories (Table 2), with a female:male ratio between 1.71 (anxiety disorders) to 13.54 (eating disorders), with a similar pattern in the set of index individuals in the analysis of cousins (supplementary Table 1). Although males were on average older when they were diagnosed with any anxiety disorder or MDD, this difference was very small (Table 2). Females were on average older when they were diagnosed with any eating disorders. Age at first diagnosis was included as a covariate in further analyses.

**Table 2:** Gender-specific sample sizes and ages at first diagnosis in the sibling analyses

Diagnostic category	Sample of unique index individuals			Age at first diagnosis, mean (s.d.)		OR (95% CIs)	*P*
	Females	Males	Female:male ratio	Females	Males		
Anxiety disorders	38 160	22 260	1.71	20.1 (5.1)	20.2 (5.7)	0.99 (0.99–1.00)	<0.0001
Major depressive disorders	36 439	21 221	1.72	20.2 (4.8)	20.6 (5.1)	0.98 (0.98–0.98)	<0.0001
Bipolar disorders	4899	2365	2.07	22.2 (4.8)	22.4 (5.6)	0.99 (0.98–1.00)	0.13
Eating disorders	15 584	1151	13.54	18.0 (3.9)	16.5 (4.5)	1.12 (1.09–1.15)	<0.0001

The four diagnostic categories were frequently comorbid, with 30.9% of the participants receiving a diagnosis from multiple categories throughout the follow-up period; see supplementary Table 2. Female patients were more likely to have multiple diagnoses than male patients (odds ratio OR = 1.65, 95% CI 1.60–1.69). The most common pair of comorbid conditions were anxiety disorders and MDD, in both genders.

### Primary analyses

Table 3 shows the results of the association analyses between the gender of the diagnosed index individual and presence of a sibling diagnosed with ADHD or broader neurodevelopmental disorders. In individuals diagnosed with anxiety disorders, female patients were more likely than the male patients to have a brother diagnosed with ADHD (OR = 1.13, 95% CI 1.05–1.22) or neurodevelopmental disorders (OR = 1.12, 95% CI 1.05–1.19). The point estimates for these comparisons were a little lower and non-significant after multiple testing correction in the analyses of sisters (ADHD: OR = 1.06, , 95% CI 0.96–1.16; neurodevelopmental disorders: OR = 1.09, 95% CI 1.01–1.18). Although the point estimates were elevated for the analysis of brothers of individuals with bipolar disorders (ADHD: OR = 1.13, 95% CI 0.94–1.40; neurodevelopmental disorders: OR = 1.11, 95% CI 0.92–1.33) and sisters of individuals with eating disorders (ADHD: OR = 1.39, 95% CI 0.92–2.10; neurodevelopmental disorders: OR = 1.33, 95% CI 0.94–1.89), these confidence intervals overlapped with 1. Thus, there were no significant associations in index individuals with MDD, bipolar disorders or eating disorders; see Table 3 for details.

**Table 3:** Results of the association between exposure to sibling with attention-deficit hyperactivity disorder (ADHD)/neurodevelopmental disorders and gender of index individual, stratified by sibling gender^a^

Diagnostic category, sibling gender	*n*^b^	ADHD			Neurodevelopmental disorders		
		OR	(95% CI)	*P*	OR	(95% CI)	*P*
Anxiety disorders							
Male	44 871	1.13	(1.05–1.22)	**0.0017**	1.12	(1.05–1.19)	**0.00053**
Female	42 704	1.06	(0.96–1.16)	0.23	1.09	(1.01–1.18)	0.023
Major depressive disorders							
Male	43 165	1.00	(0.93–1.08)	0.98	0.99	(0.93–1.05)	0.72
Female	40 673	1.03	(0.94–1.13)	0.55	1.08	(1.00–1.17)	0.05
Bipolar disorders							
Male	5341	1.13	(0.91–1.40)	0.28	1.11	(0.92–1.33)	0.28
Female	5043	1.08	(0.84–1.39)	0.53	1.07	(0.87–1.32)	0.51
Eating disorders							
Male	12 124	0.90	(0.66–1.23)	0.52	0.85	(0.66–1.09)	0.19
Female	11 579	1.39	(0.92–2.10)	0.12	1.33	(0.94–1.89)	0.11

a. Gender of the index individual is coded as male, 0 and female, 1. All estimates are obtained from models adjusted for covariates, as described in text. Bonferroni corrected *P*-value threshold: *P* < 0.0031. Results that are significant at this threshold are in bold.

b. Includes all sibling-index pair observations.

### Secondary analyses

When the main analyses were repeated for exposure to risk of broad neurodevelopmental disorders not including ADHD, the effect sizes decreased, the confidence intervals increased and there were no associations for any of the diagnostic categories (supplementary Table 3). Thus, all further analyses focused on just ADHD diagnosis as the exposure.

Stratifying the index individuals based on presence of comorbid ADHD yielded significant associations between having a brother with ADHD and female anxiety in those with comorbid ADHD (*n* = 7327, OR = 1.20, 95% CI 1.05–1.37), *P* = 0.0066], with a smaller, non-significant effect in those without comorbid ADHD (*n* = 37 544, OR = 1.09, 95% CI 1.00–1.20, *P* = 0.061). In contrast, the analysis of sisters with ADHD showed no association with gender in index individuals with anxiety and comorbid ADHD (*n* = 7104, OR = 0.94, 95% CI 0.81–1.10, *P* = 0.47), yet an association in those without comorbid ADHD (*n* = 35 600, OR = 1.14, 95% CI 1.01–1.29, *P* = 0.031). No associations were seen for the other disorders (supplementary Table 4).

**Fig. 1:**
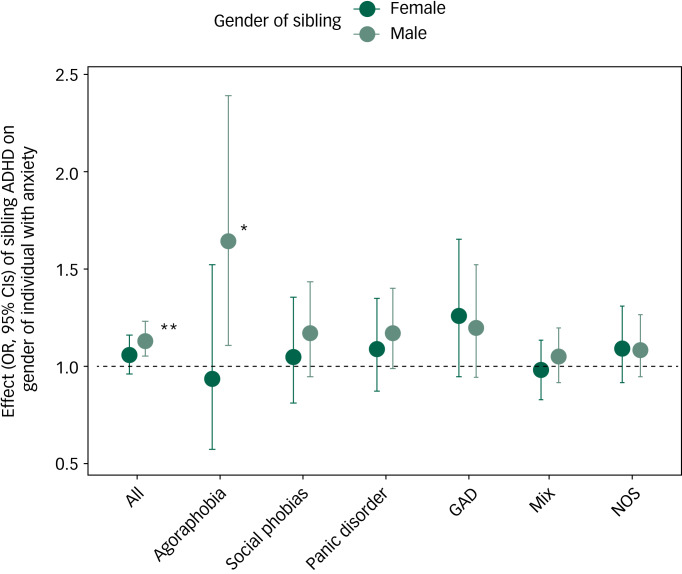
Results of association between exposure to a sibling being diagnosed with attention-deficit hyperactivity disorder and gender of index individuals with different categories of anxiety disorders.

GAD, generalised anxiety disorder; Mix, mixed anxiety and depressive disorder; NOS, not otherwise specified. The category NOS excludes individuals if diagnosed with any of the other categories; otherwise the categories are not mutually exclusive. *P < 0.05, **P < 0.01.

The primary association seen in the context of anxiety was followed up to determine whether any specific type(s) of anxiety disorders were driving the association signal. Supplementary Table 5 displays detailed numbers for the ICD-10 anxiety categories. The following ICD codes, each affecting at least 3% of the individuals with anxiety, were examined: agoraphobia (F40.0), social phobias (F40.1), panic disorder (F41.0), generalised anxiety disorder (GAD; F41.1), mixed anxiety and depressive disorder (F41.2) and unspecified anxiety disorder (F41.9). The latter category was only considered as present if none of the other categories of anxiety examined were present. Compared with the primary analyses, these follow-up analyses showed similar point estimates for several categories (i.e. agoraphobia, social phobias, panic disorder and GAD) in the analyses of brothers, albeit the confidence intervals overlapped with 1 for most categories, except agoraphobia: OR = 1.64, 95% CI 1.12–2.39; see Fig. 1 and supplementary Table 6 for details.

The estimated magnitude of effect sizes for the association between having a cousin with ADHD and the gender of index individuals with anxiety were lower than seen in the sibling-pair analyses and non-significant (OR, , 95% CI  1.00, 95% CI 0.96–1.05). No other associations were significant in the cousin analyses (supplementary Table 7).

### Sensitivity analyses

Sensitivity analyses were used to examine the stability of the results; see supplementary Table 1 for sample sizes and female:male ratios. Of note, the exclusions for these analyses were biased towards excluding more index males (in sensitivity analyses 1 and 2), as evidenced by comparing the sample sizes and female:male ratios in Table 2 and supplementary Table 1.

Supplementary Table 8 displays the results of the sensitivity analyses aiming to account for diagnostic uncertainty. The estimated association between female anxiety and having a male sibling with ADHD was lower with a wider confidence interval than in the primary analysis (OR = 1.05, 95% CI 0.95–1.17).

Restricting the analyses to observations where both individuals were aged ≥18 years old at the end of the follow-up period (74.5% of the sample of unique index individuals) also decreased this association compared with the primary analyses (OR = 1.09, 95% CI 0.99–1.19) (supplementary Table 9). Although the confidence intervals still overlapped with 1 for the association between females with bipolar disorders and having a brother with ADHD, the point estimate increased slightly when analysing only adults (OR = 1.22, 95% CI 0.94–1.57), compared with the full analysis (Table 3).

Excluding observations with incomplete information during follow-up did not substantially affect the results (supplementary Table 10). The sensitivity analyses did not reveal any consistent patterns of association results in individuals diagnosed with MDD, bipolar or eating disorders.

## Discussion

The aim of this study was to test the hypothesis that female patients diagnosed with anxiety, depressive, bipolar and eating disorders are more likely to have a relative with ADHD or another neurodevelopmental disorder compared with affected male patients. We found modest support for this hypothesis in individuals diagnosed with anxiety disorders. Although the effect sizes in the analyses of brothers of those with bipolar disorders and sisters of those with eating disorders were similar to those observed for anxiety, they were statistically non-significant, perhaps because of the lower power associated with the lower prevalence of these disorders. No associations were seen for MDD, despite a similar sample size to that available for anxiety disorders.

The results observed for anxiety are consistent with two studies of Swedish children, which found that females with anxiety and/or depression had a higher burden from genetic variants implicated in ADHD.^10,11^ Notably, there were relatively few MDD diagnoses in these previous studies, because of the young sample. In the current study, we find supporting evidence in adults. Although non-significant, the point estimates in the sensitivity analyses of only adults were not that different for anxiety (OR = 1.09; 74.6% of sample) and MDD (OR = 1.04; 78.1% of sample) and higher for bipolar disorders (OR = 1.22; 85.1% of sample), suggesting that differences in age at diagnosis could explain the discrepant results observed for anxiety and MDD in the full cohort.

Our non-significant results for MDD are inconsistent with a recent UK population study of middle-aged adults, which found weak evidence to suggest that rare neuropsychiatric copy number variants may be associated with depression in females.^25^ On the other hand, another study of this UK population found no gender interaction with polygenic risk scores for ADHD and risk of anxiety, MDD and bipolar disorders,^26^ which is consistent with our results for MDD and bipolar disorders but inconsistent with the results for anxiety. Our results suggest that the age of diagnosed individuals may have an impact on the observation of an association between familial/genetic risks and gender differences in psychiatric disorders.

Follow-up analyses for the association between ADHD in brothers and anxiety in females revealed broadly consistent estimates for several categories of anxiety, albeit only the association in agoraphobia was statistically significant. Although agoraphobia typically onsets in early adulthood, it may be preceded by transient and subthreshold childhood anxiety symptoms.^3^ Further work is needed to determine whether only certain anxiety subtypes show gender-specific associations with ADHD risk.

The association in individuals with anxiety was seen for observations from brothers but not sisters. This may be because sisters are less likely to be diagnosed with ADHD, therefore, decreasing the likelihood of observing the same association as seen in the analysis of brothers. However, in index individuals without comorbid ADHD, the estimate for the association between ADHD in sisters and female anxiety increased. In contrast, there was a larger estimated effect of brother's ADHD on female gender in those with comorbid ADHD, with a smaller effect in those without ADHD. This suggests that gender-specific effects may differ depending on the presence of comorbid diagnoses.

The observed association in anxiety was not significant in the cousin-pair analyses, despite the larger sample size. We could not account for the possibility that shared environmental effects or gene × environment correlations also contributed (for example via disorder manifestation or likelihood of clinical referral). However, twin studies have shown a limited role for shared environmental effects in ADHD,^27^ suggesting that genetic factors are likely to be of greater importance in explaining the familial effect we observed. Thus, the results imply that in individuals diagnosed with anxiety disorders, females are more likely to be at familial (likely genetic) risk for ADHD than males, consistent with previously observed molecular genetic effects.^10,11^

One possible interpretation of these results is that there is gender-specific manifestation of risk factors, where females at genetic risk for psychiatric problems may be more likely to develop, or be diagnosed with, anxiety rather than ADHD or both anxiety and ADHD, whereas at-risk males may be more likely to have ADHD than anxiety. The results suggest that comorbid ADHD and anxiety in females may also be linked to familial risk of ADHD. Although our stratified analyses suggested that the association between brother's ADHD and female anxiety was somewhat stronger in individuals with comorbid ADHD, the confidence intervals overlapped with those in individuals without comorbid ADHD and the opposite pattern was seen for sisters. In previous work, accounting for parent-reported ADHD symptoms did not explain the increased ADHD genetic burden in females with anxiety and depression.^10,11^

The mechanisms for how different psychopathology could emerge in males and females are unknown but could potentially involve hormonal differences and/or gender-specific parenting and social influences. Alternatively, there could be gender-specific diagnostic biases in clinical practice, such as diagnostic overshadowing, where females at genetic risk for psychiatric problems who are presenting with complex psychopathology may be more likely to receive a diagnosis of anxiety instead of ADHD or both anxiety and ADHD, whereas males may be more likely to be diagnosed with ADHD. In this case, females being diagnosed with anxiety could indeed have symptoms of ADHD that are either being missed, do not meet diagnostic symptom criteria or are only diagnosed later. Indeed, in the group of index individuals with comorbid anxiety and ADHD, females were more likely than males to be diagnosed with anxiety before ADHD (55.9% of females *v.* 43.5% of males). Also, this is plausible given that inattentive ADHD symptoms (which are likely to be less easily identified than hyperactive symptoms) are more common in females^28,29^ and the inattentive subtype of ADHD may be underdiagnosed when following ICD criteria. Also, the current diagnostic criteria for ICD and DSM are primarily based on symptoms typically observed in males and may therefore miss symptoms more characteristic of female ADHD.^30,31^ It is notable that in index individuals with comorbid ADHD and anxiety in our study, females were diagnosed with ADHD later than males (females: mean 19.2 (s.d. = 5.3) years; males: mean 18.3 (s.d. = 6.4) years; OR = 1.03, 95% CI 1.02–1.03).

To address the possibility of diagnostic biases, we excluded individuals with possible diagnostic uncertainty. This attenuated the observed effect, which indicates a likely role for gender-specific biases in clinical practice, which resolve over time. However, these stringent diagnostic exclusions also decreased the sample size and statistical power, which may also contribute to the weaker results. Thus, we are unable to definitively conclude whether our results are best accounted for by gender-specific manifestation of risks or gender biases in clinical practice, but these explanations are not necessarily mutually exclusive.

Our results examining a broader group of neurodevelopmental disorders showed that the observed effect was limited to when ADHD was included in the definition of neurodevelopmental disorders. This suggests that the results may not apply to neurodevelopmental disorders beyond ADHD, despite shared genetic risks reported across these disorders.^9^ However, the coverage of several ICD diagnoses (such as communication disorders, specific intellectual disorders and dyslexia) is incomplete in the register as these conditions are commonly diagnosed by educational psychologists. This, together with the lower sample size for neurodevelopmental disorders beyond ADHD may have had an impact on the observed results. Further work is needed to determine whether the results of this study extend to a broader range of neurodevelopmental disorders.

### Limitations

The results need to be interpreted in light of several limitations. Our effect sizes are rather modest. Furthermore, despite including all individuals in Sweden diagnosed with ICD-10 clinical diagnoses of anxiety disorders, MDD, bipolar and eating disorders, there were relatively few individuals, particularly males, with bipolar disorders and eating disorders. As a result of the use of real-life clinical data, we may have missed individuals who are affected with psychiatric disorders but do not have a recorded diagnosis in the register; as such, our results relate to patterns of diagnoses in individuals who come to clinical attention, who may be the most severely affected. To minimise the possibility of missing information on ADHD in relatives, we applied more stringent exclusion criteria, and this did not substantially have an impact on the results.

Our inclusion and exclusion criteria, particularly for several of the sensitivity analyses, resulted in more index males being excluded, which may have had an impact on the representativeness of the data. Finally, because of the selection of the primary cohort on the basis of having full siblings, the results are not representative of individuals with no siblings; therefore, stoppage effects, which may operate when parents do not have further children (for example in the presence of psychiatric difficulties), could have an important impact that we were not able to investigate as part of this study design. Ultimately, a variety of study designs will be needed to determine whether and to what extent gender-specific manifestation of psychiatric risks and gender-specific diagnostic biases may explain the widespread gender differences in prevalence of psychiatric disorders.

### Implications

In short, we report that females with diagnosed anxiety are more likely to have a brother with ADHD, compared with males diagnosed with anxiety. This suggests that familial/genetic risk factors for ADHD may be more likely to manifest as anxiety in females compared with males. This association between familial risk for ADHD and gender of the person affected by a psychiatric disorder does not surpass statistical significance in the context of depression, bipolar disorders or eating disorders, showing limited support for gender-specific effects. Our results support previous work by suggesting that gender-specific manifestation of familial/genetic risk or gender-specific diagnostic biases may play a small role in explaining the gender differences seen in the prevalence of ADHD and anxiety. Further research using molecular genetic approaches will be needed to confirm and extend these results. The results highlight the importance of studying gender differences in prevalence to better understand psychiatric disorders with a view towards improving clinical practice to more effectively target interventions to diagnose and help affected individuals.

## Data Availability

J.M., L.G. and Q.C. have ongoing access to the study data.

## Supplementary material

For supplementary material accompanying this paper visit http://dx.doi.org/10.1192/bjo.2020.47. click here to view supplementary material
