# Supplementary material for: Investigating gender-specific effects of familial risk for attention-deficit hyperactivity disorder and other neurodevelopmental disorders in the Swedish population
Source: BJPsych Open. 2020 Jun 18;6(4):e65. doi: 10.1192/bjo.2020.47 (PMC7345736; doi:10.1192/bjo.2020.47)
Supplement: Supplementary file 1 [file bjosup.zip › S2056472420000472sup001.docx]

# Supplemental Materials

**Supplementary Table 1: Number of index individuals in cousin-pair & sensitivity analyses**

| **Analysis** | **Diagnostic category** | **Sample of unique index individuals** | | |
| --- | --- | --- | --- | --- |
|  |  | **Females** | **Males** | **F:M ratio** |
| **Cousins** | **AD** | 63201 | 37629 | 1.68 |
|  | **MDD** | 62566 | 37217 | 1.68 |
|  | **BD** | 9612 | 4975 | 1.93 |
|  | **ED** | 20786 | 1356 | 15.33 |
| **Sensitivity analysis 1: All diagnoses must be present at least twice** | **AD** | 23159 | 12674 | 1.83 |
|  | **MDD** | 24433 | 13726 | 1.78 |
|  | **BD** | 3781 | 1762 | 2.15 |
|  | **ED** | 11798 | 749 | 15.75 |
| **Sensitivity analysis 2: Only individuals aged ≥18 years old** | **AD** | 28616 | 16435 | 1.74 |
|  | **MDD** | 28460 | 16581 | 1.72 |
|  | **BD** | 4205 | 1977 | 2.13 |
|  | **ED** | 11118 | 676 | 16.45 |
| **Sensitivity analysis 3:**  **Stringent exclusion criteria in siblings** | **AD** | 35531 | 20472 | 1.74 |
|  | **MDD** | 33844 | 19545 | 1.73 |
|  | **BD** | 4574 | 2176 | 2.10 |
|  | **ED** | 14599 | 1077 | 13.56 |

AD: anxiety disorders; MDD: major depressive disorders; BD: bipolar disorders; ED: eating disorders.

## Supplementary Table 2: Patterns of single and multiple diagnoses in index individuals

| **Comorbid diagnoses** | **Females** | | **Males** | | **Total sample** | |
| --- | --- | --- | --- | --- | --- | --- |
|  | **N** | **%** | **N** | **%** | **N** | **%** |
| **AD-only** | 18,840 | 28.20 | 13,962 | 37.61 | 32,802 | 31.56 |
| **MDD-only** | 15,816 | 23.67 | 12,652 | 34.08 | 28,468 | 27.39 |
| AD + MDD | 12,445 | 18.63 | 7,030 | 18.94 | 19,475 | 18.74 |
| **ED-only** | 8,093 | 12.11 | 671 | 1.81 | 8,764 | 8.43 |
| MDD + ED | 2,637 | 3.95 | 156 | 0.42 | 2,793 | 2.69 |
| AD + MDD + ED | 2,474 | 3.70 | 154 | 0.41 | 2,628 | 2.53 |
| AD + MDD + BD | 1,542 | 2.31 | 638 | 1.72 | 2,180 | 2.10 |
| **BD-only** | 951 | 1.42 | 809 | 2.18 | 1,760 | 1.69 |
| AD + ED | 1,611 | 2.41 | 136 | 0.37 | 1,747 | 1.68 |
| MDD + BD | 931 | 1.39 | 567 | 1.53 | 1,498 | 1.44 |
| AD + BD | 706 | 1.06 | 317 | 0.85 | 1,023 | 0.98 |
| AD + MDD + BD + ED | 451 | 0.67 | 19 | 0.05 | 470 | 0.45 |
| MDD + BD + ED | 143 | 0.21 | 5 | 0.01 | 148 | 0.14 |
| AD + BD + ED | 91 | 0.14 | 4 | 0.01 | 95 | 0.09 |
| BD + ED | 84 | 0.13 | 6 | 0.02 | 90 | 0.09 |
| **Total with single disorder** | 43,700 | 65.40 | 28,094 | 75.68 | 71,794 | 69.07 |
| **Total with multiple disorders** | 23,115 | 34.60 | 9,032 | 24.33 | 32,147 | 30.93 |

Table sorted by Total sample N. AD: anxiety disorders; MDD: major depressive disorders; BD: bipolar disorders; ED: eating disorders.

## Supplementary Table 3: Results of association between exposure to sibling with neurodevelopmental disorders, not including ADHD

| **Diagnostic category** | **Sibling sex** | **N** | **OR** | **LCI-UCI** | **p** |
| --- | --- | --- | --- | --- | --- |
| **AD** | **Male** | 44871 | 1.01 | (0.91-1.11) | 0.90 |
|  | **Female** | 42704 | 1.05 | (0.93-1.19) | 0.41 |
| **MDD** | **Male** | 43165 | 0.91 | (0.82-1.01) | 0.073 |
|  | **Female** | 40673 | 1.08 | (0.94-1.23) | 0.27 |
| **BD** | **Male** | 5341 | 1.01 | (0.75-1.36) | 0.93 |
|  | **Female** | 5043 | 0.97 | (0.68-1.37) | 0.84 |
| **ED** | **Male** | 12124 | 0.73 | (0.50-1.05) | 0.090 |
|  | **Female** | 11579 | 1.02 | (0.60-1.73) | 0.94 |

Sex of the index individual is coded as male=0 and female=1. All estimates are obtained from models adjusted for covariates, as described in text.

AD: anxiety disorders; MDD: major depressive disorders; BD: bipolar disorders; ED: eating disorders.

## Supplementary Table 4: Results of the association between exposure to sibling with ADHD and sex of index individual, stratified by presence/absence of comorbid ADHD in index individuals

| **Diagnostic category** | **Sibling sex** | **Comorbid ADHD** | | | | **No comorbid ADHD** | | | |
| --- | --- | --- | --- | --- | --- | --- | --- | --- | --- |
|  |  | **N** | **OR** | **LCI-UCI** | **p** | **N** | **OR** | **LCI-UCI** | **p** |
| **AD** | **Male** | 7327 | 1.20 | (1.05-1.37) | 6.6E-03 | 37544 | 1.09 | (1.00-1.20) | 0.061 |
|  | **Female** | 7104 | 0.94 | (0.81-1.10) | 0.47 | 35600 | 1.14 | (1.01-1.29) | 0.031 |
| **MDD** | **Male** | 7021 | 1.00 | (0.87-1.14) | 0.99 | 36144 | 1.01 | (0.91-1.11) | 0.90 |
|  | **Female** | 6671 | 1.00 | (0.85-1.17) | 0.99 | 34002 | 1.05 | (0.93-1.18) | 0.42 |
| **BD** | **Male** | 1413 | 1.08 | (0.78-1.49) | 0.64 | 3928 | 1.19 | (0.88-1.61) | 0.26 |
|  | **Female** | 1318 | 0.95 | (0.66-1.37) | 0.79 | 3725 | 1.24 | (0.87-1.78) | 0.23 |
| **ED** | **Male** | 848 | 0.91 | (0.53-1.55) | 0.72 | 11276 | 0.90 | (0.62-1.31) | 0.58 |
|  | **Female** | 829 | 1.07 | (0.57-1.99) | 0.84 | 10750 | 1.60 | (0.91-2.81) | 0.11 |

Sex of the index individual is coded as male=0 and female=1. All estimates are obtained from models adjusted for covariates, as described in text.

AD: anxiety disorders; MDD: major depressive disorders; BD: bipolar disorders; ED: eating disorders.

## Supplementary Table 5: Sex-specific sample sizes for different ICD categories of anxiety disorders

| **Diagnostic category** | **ICD code** | **% of those with any AD** | **Sample of unique index individuals** | | |
| --- | --- | --- | --- | --- | --- |
|  |  |  | **Females** | **Males** | **F:M ratio** |
| **Agoraphobia** | F40.0 | 3.6% | 1368 | 801 | 1.71 |
| **Social phobias** | F40.1 | 11.9% | 4132 | 3079 | 1.34 |
| **Specific phobias** | F40.2 | 2.7% | 1094 | 529 | 2.07 |
| **Other phobic ADs** | F40.8 | 0.4% | 178 | 91 | 1.96 |
| **Phobic AD-NOS** | F40.9 | 1.3% | 442 | 369 | 1.20 |
| **Panic disorder** | F41.0 | 19.5% | 7656 | 4135 | 1.85 |
| **Generalized AD** | F41.1 | 10.6% | 4208 | 2202 | 1.91 |
| **Mixed anxiety and depressive disorder** | F41.2 | 33.4% | 13887 | 6300 | 2.20 |
| **Other mixed ADs** | F41.3 | 1.3% | 510 | 272 | 1.88 |
| **Other specified ADs** | F41.8 | 1.6% | 584 | 389 | 1.50 |
| **AD-NOS** | F41.9 | 48.9% | 18605 | 10941 | 1.70 |
| **AD NOS, with exclusions*** | F41.9 | 31.1% | 11366 | 7425 | 1.53 |
| **Separation AD of childhood** | F93.0 | 0.9% | 268 | 298 | 0.90 |
| **Phobic AD of childhood** | F93.1 | 0.3% | 80 | 75 | 1.07 |
| **Social AD of childhood** | F93.2 | 0.4% | 123 | 142 | 0.87 |
| **Sibling rivalry disorder** | F93.3 | 0.2% | 52 | 74 | 0.70 |
| **Other childhood emotional disorders** | F93.8 | 0.6% | 227 | 152 | 1.49 |
| **Childhood emotional disorder NOS** | F93.9 | 0.9% | 326 | 233 | 1.40 |

AD: anxiety disorder; NOS: not otherwise specified. Disorder categories affecting more than 3% of the sample are highlighted in grey; these categories were analysed further.

* Excludes individuals if diagnosed with F40.0, F41.0, F41.0, F41.1, or F41.2. Note that otherwise the categories are not mutually exclusive.

## Supplementary Table 6: Results of association between exposure to a sibling diagnosed with ADHD and sex of index individuals diagnosed with different categories of anxiety disorders

| **Diagnostic category** | **Sibling sex** | **N** | **OR** | **LCI-UCI** | **p** |
| --- | --- | --- | --- | --- | --- |
| **Agoraphobia** | **Male** | 1555 | 1.64 | (1.12-2.39) | 0.011 |
|  | **Female** | 1498 | 0.93 | (0.58-1.51) | 0.78 |
| **Social phobias** | **Male** | 5121 | 1.17 | (0.95-1.43) | 0.13 |
|  | **Female** | 4958 | 1.05 | (0.82-1.35) | 0.68 |
| **Panic disorder** | **Male** | 8816 | 1.17 | (0.99-1.40) | 0.070 |
|  | **Female** | 8430 | 1.09 | (0.87-1.35) | 0.45 |
| **GAD** | **Male** | 4542 | 1.20 | (0.95-1.52) | 0.13 |
|  | **Female** | 4329 | 1.25 | (0.94-1.65) | 0.13 |
| **Mixed** | **Male** | 14985 | 1.04 | (0.92-1.19) | 0.52 |
|  | **Female** | 14137 | 0.98 | (0.83-1.14) | 0.76 |
| **AD-NOS*** | **Male** | 14182 | 1.09 | (0.95-1.26) | 0.22 |
|  | **Female** | 13324 | 1.10 | (0.93-1.30) | 0.25 |

Mixed: mixed anxiety and depressive disorder; GAD: generalized anxiety disorder; AD-NOS: anxiety disorder, not otherwise specified.

* Excludes individuals if diagnosed with F40.0, F41.0, F41.0, F41.1, or F41.2. Note that otherwise the categories are not mutually exclusive.

**Supplementary Table 7: Results of association between exposure to a cousin being diagnosed with ADHD and sex of index individuals, stratified by cousin sex**

| **Diagnostic category** | **Cousin sex** | **N-obs** | **OR** | **LCI-UCI** | **p** |
| --- | --- | --- | --- | --- | --- |
| **AD** | **Male** | 195831 | 1.00 | (0.96-1.05) | 0.95 |
|  | **Female** | 186209 | 1.02 | (0.96-1.08) | 0.47 |
| **MDD** | **Male** | 190943 | 1.02 | (0.98-1.07) | 0.28 |
|  | **Female** | 181254 | 0.99 | (0.93-1.05) | 0.77 |
| **BD** | **Male** | 26869 | 1.04 | (0.93-1.17) | 0.48 |
|  | **Female** | 25500 | 1.10 | (0.94-1.29) | 0.23 |
| **ED** | **Male** | 45607 | 0.99 | (0.82-1.20) | 0.92 |
|  | **Female** | 43121 | 1.10 | (0.86-1.42) | 0.44 |

Sex of the index individual is coded as male=0 and female=1. All estimates are obtained from models adjusted for covariates, as described in text.

AD: anxiety disorders; MDD: major depressive disorders; BD: bipolar disorders; ED: eating disorders; ADHD: attention deficit hyperactivity disorder.

## Supplementary Table 8: Results of sensitivity analyses in sibling pair observations, considering all diagnoses as present only if they were received at least twice

| **Diagnostic category** | **Sibling sex** | **N-obs** | **OR** | **LCI-UCI** | **p** |
| --- | --- | --- | --- | --- | --- |
| **AD** | **Male** | 26058 | 1.05 | (0.95-1.17) | 0.32 |
|  | **Female** | 24870 | 0.99 | (0.87-1.12) | 0.89 |
| **MDD** | **Male** | 28216 | 1.02 | (0.92-1.13) | 0.73 |
|  | **Female** | 26839 | 0.99 | (0.87-1.12) | 0.86 |
| **BD** | **Male** | 4010 | 1.06 | (0.81-1.39) | 0.66 |
|  | **Female** | 3852 | 1.07 | (0.78-1.45) | 0.69 |
| **ED** | **Male** | 8995 | 1.07 | (0.68-1.67) | 0.77 |
|  | **Female** | 8649 | 1.09 | (0.65-1.83) | 0.75 |

Sex of the index individual is coded as male=0 and female=1. All estimates are obtained from models adjusted for covariates, as described in text.

AD: anxiety disorders; MDD: major depressive disorders; BD: bipolar disorders; ED: eating disorders; ADHD: attention deficit hyperactivity disorder.

## Supplementary Table 9: Results of sensitivity analyses in sibling pair observations, restricting to individuals who are 18 years or older at the end of the follow-up period

| **Diagnostic category** | **Sibling sex** | **N-obs** | **OR** | **LCI-UCI** | **p** |
| --- | --- | --- | --- | --- | --- |
| **AD** | **Male** | 32111 | 1.09 | (0.99-1.19) | 0.075 |
|  | **Female** | 30515 | 1.04 | (0.93-1.16) | 0.50 |
| **MDD** | **Male** | 32276 | 1.04 | (0.95-1.14) | 0.42 |
|  | **Female** | 30513 | 1.00 | (0.90-1.11) | 0.98 |
| **BD** | **Male** | 4314 | 1.22 | (0.94-1.57) | 0.13 |
|  | **Female** | 4163 | 1.06 | (0.81-1.40) | 0.66 |
| **ED** | **Male** | 8228 | 1.05 | (0.69-1.61) | 0.82 |
|  | **Female** | 7772 | 1.23 | (0.75-2.03) | 0.41 |

Sex of the index individual is coded as male=0 and female=1. All estimates are obtained from models adjusted for covariates, as described in text.

AD: anxiety disorders; MDD: major depressive disorders; BD: bipolar disorders; ED: eating disorders; ADHD: attention deficit hyperactivity disorder.

## Supplementary Table 10: Results of sensitivity analyses in sibling pair observations, using stringent exclusion criteria in relatives

| **Diagnostic category** | **Sibling sex** | **N-obs** | **OR** | **LCI-UCI** | **p** |
| --- | --- | --- | --- | --- | --- |
| **AD** | **Male** | 39886 | 1.13 | (1.04-1.22) | 2.5E-03 |
|  | **Female** | 38022 | 1.05 | (0.95-1.15) | 0.32 |
| **MDD** | **Male** | 38358 | 0.97 | (0.90-1.05) | 0.48 |
|  | **Female** | 36185 | 1.00 | (0.91-1.10) | 0.96 |
| **BD** | **Male** | 4835 | 1.16 | (0.93-1.45) | 0.20 |
|  | **Female** | 4524 | 1.05 | (0.81-1.35) | 0.74 |
| **ED** | **Male** | 11012 | 0.88 | (0.64-1.20) | 0.42 |
|  | **Female** | 10510 | 1.39 | (0.91-2.13) | 0.13 |

Sex of the index individual is coded as male=0 and female=1. All estimates are obtained from models adjusted for covariates, as described in text.

AD: anxiety disorders; MDD: major depressive disorders; BD: bipolar disorders; ED: eating disorders; ADHD: attention deficit hyperactivity disorder.
